# Is remaining intervertebral disc tissue interfering with bone generation during fusion of two vertebrae?

**DOI:** 10.1371/journal.pone.0215536

**Published:** 2019-04-25

**Authors:** D. Kok, C. M. M. Peeters, Z. Mardina, D. L. M. Oterdoom, S. K. Bulstra, A. G. Veldhuizen, R. Kuijer, F. H. Wapstra

**Affiliations:** 1 Department of Orthopaedics, Rijnstate Hospital, Arnhem, The Netherlands; 2 Department of Orthopaedics, University of Groningen, University Medical Center Groningen, Groningen, The Netherlands; 3 Department of Biomedical Engineering, University of Groningen, University Medical Center Groningen, Groningen, The Netherlands; 4 Department of Neurosurgery, University of Groningen, University Medical Center Groningen, Groningen, The Netherlands; Mayo Clinic Minnesota, UNITED STATES

## Abstract

**Study design:**

laboratory research.

**Background:**

Through the increasing number of minimally invasive procedures in spinal fusion surgery, the complete removal of intervertebral disc (IVD) tissue has become more a challenge. Remaining IVD may interfere with the biological process of bone formation.

**Objective:**

In order to establish whether complete removal of IVD tissue will improve or inhibit the fusion process, the effects of different concentrations of extracts of inflamed disc tissue on the mitochondrial activity of mesenchymal stem cells (MSCs), and the capacity to mineralize their extracellular matrix by osteoblasts and differentiated MSCs were tested *in vitro*.

**Methods:**

A MTT assay was conducted to measure the mitochondrial activity of MSCs, and an Alizarin Red S staining quantification assay to measure the deposition of calcium by osteoblasts and differentiated, bone marrow-derived MSCs.

**Results:**

A significantly higher mitochondrial activity was shown in MSCs co-cultured with extracts of IVD tissue (10%, 50%, and 100%) compared with the control group after 48 hours of incubation, indicating that the IVD tissue extracts stimulated the mitochondrial activity of MSCs. This effect appeared to be inversely proportional to the concentration of IVD tissue extract. No significant differences in mineralization by human osteoblasts or differentiated MSCs were found between the samples incubated with IVD tissue extracts (3% and 33%) and the control samples.

**Conclusion:**

Our findings indicate that remaining IVD tissue has more of a stimulating than inhibiting effect on the activity of MSCs. Even if inflammatory cytokines are produced, these do not result in a net inhibition of cellular activity or osteogenic differentiation of MSCs.

## Introduction

The number of spinal fusion surgeries has been increased considerably in the last few decades[1]. For a solid fusion of two vertebrae it is essential to perform a discectomy and remove the tissue of the intervertebral disc (IVD) as well as both the vertebral endplate cartilages[2]. However, during minimally invasive procedures using the transforaminal lumbar interbody fusion technique (TLIF), the complete removal of intervertebral tissue and the vertebral cartilages is a challange[2,3].

Remaining disc tissue may interfere with the biological process of bone formation, and so increase the likelihood of a poor fusion of the vertebrae. In particular nucleus pulposus (NP) tissue is described to have inflammatory properties and secretes cytokines which may intervene in the metabolism of mesenchymal stem cell (MSC) or osteoblasts[4,5]. Since osteoblasts are responsible for the synthesis of the bone matrix, and MSCs have the ability to differentiate towards osteoblasts, these cells play an crucial role in the bone formation process[6]. It is well known that inflammation is necessary to regulate MSC osteogenesis[7]. The environment of MSCs has been shown to fulfill an important role in behavior of the cells and differentiation processes[7]. The exact interplay between the presence and concentrations of different cytokines and their influence on MSC behaviour, remains unknown. The presence of inflammatory cytokines secreted by remaining IVD tissue could for example decrease the cell viability of MSCs or interfere with the differentiation of MCSs to osteoblasts. Indirectly, this would have a negative impact on bone matrix formation. Besides possible interactions with MSCs, the cytokines of remaining IVD tissue could also inhibit directly osteoblast activity, and in this way result in less bone matrix formation. In order to establish whether complete removal of IVD tissue will improve the fusion process, we have tested the effects of different concentrations of inflamed disc tissue extracts on the viabiliy of MSCs (i), and the matrix production of osteoblasts (ii), and differentiated MSCs (iii) *in vitro*.

## Methods

### Preparation of tissue extraxts

Nucleus pulposis (NP) and annulus fibrosus (AF) tissues were obtained from six random patients who underwent posterior lumbar interbody fusion (PLIF) surgery at the University Medical Center Groningen, after informed consent of the patients and according to the legal procedures for the use of to be discarded body tissue for experimental research. The Medical Ethical Committee of the University Medical Center Groningen had approved the use of removed tissue samples for scientific purposes, provided the samples were anonymised. The mean age of the patients was 56.5 and ranged from 30 to 70 years old (Table 1). The male to female ratio was 1:1. Tissues were transferred to the laboratory in pre-weighed sterile vials filled with transport medium consisting of Dulbecco’s Modified Eagle’s Medium (DMEM)-high glucose (Life Technology, Bleiswijk, The Netherlands) supplemented with 2% antibiotics, 0.2 mM ascorbic acid-2-phosphate and 10% fetal bovine serum (FBS). In the laboratory, the vials were weighed again to establish the weight of the collected tissue. The mean weight of tissue was 3.01 g. The tissues were washed once with phosphate buffered saline (PBS) and twice with α-MEM complete medium consisting of 90% α-MEM (Life Technology), 10% of FBS, 1% of antibiotics, and 0.2 mM of ascorbic acid-2-phosphate. Subsequently, this α-MEM complete culture medium was added to the disc tissue samples up a 5% suspension (5g / 100 ml). The tissues were extracted at 37°C in a humidified atmosphere of 5% CO_2_ and 95% air for 24 hours. After that, the extract suspensions were centrifuged at 1500 rpm for 15 minutes. The supernatants were collected and frozen at -20°C until further testing. The mean obtained extract volume was 15.6 ml.

**Table 1:** Patient characteristics.

Patient/extract	Gender	Age (y)	Weight of tissue (gram)	Obtained Extract (ml)
1	M	57	3.3357	16.6785
2	M	30	3.6362	18.1810
3	M	54	1.7919	8.9595
4	F	70	4.9388	24.6940
5	F	66	3.5960	19.6900
6	F	62	1.0485	5.2425

Abbreviations: F, female; M, male

### Human mesenchymal stem cell culture

Human MCSs were obtained from the MSC bank, containing MSCs isolated from bone marrow obtained from patients during total hip or knee replacement. The MSC bank was set up by Arina Buizer in our laboratory, who characterized all samples according to the guidelines of the International Society of Cellular Therapy (S1 File) [8]. MCSs of the third passage (P-3) were cultured in T75 flasks in α-MEM complete culture medium until 50–60% confluence at 37°C in a humidified atmosphere at 5% CO_2_. The cells were counted with a Bürker-Türk haemocytometer using a Leica inverted phase-contrast microscope (Leica DMIL LED, Leica Microsystems, Rijswijk, The Netherlands).

### Human osteoblast culture

Primary human osteoblasts, previously isolated from bone chips derived from femoral heads of patients at total hip surgery were isolated according to the procedure described by Gartland et al. (9). Briefly, femoral heads removed during total hip surgery and obtained from the operating room were transferred to the laboratory in sterile DMEM-F12 culture medium(Gibco-Life Technology), supplemented with 2% anti-anti. In the lab the bone was crushed using bone mills and bone marrow was removed during an incubation in collagenase type II solution for 24 hours. The bone chips were rinsed five times in DMEM/F12 supplemented with 2% anti-anti and then placed in cell culture flasks in culture medium. Medium was refreshed twice weekly. Cells that grew out of the bone chips were harvested when the cultures were 70% confluent. Cells were passaged and then frozen in liquid nitrogen, using standard cells culture procedures. Osteoblasts were characterized by assessment of alkaline phosphatase activity (Leukocyte alkaline phosphatase kit, Sigma, Steinheim, Germany) according to the manufacturer’s instructions. Osteoblasts were used at their third passage (P-3). The culture medium consisted for 88% of DMEM-F12 (1:1), 10% of FBS, 2% of anti-anti, and 0.2 mM of L-Ascorbic acid 2-phosphate. The osteoblasts were cultured in T75 flasks until 60% confluence.

### Influence of intervertebral disc tissue extracts on viability of MCSs

To evaluate the influence of extracts of IVD tissue on the mitochondrial activity of MSCs first a MTT assay was conducted, according to protocol BME-I-R-002 of the department of the department of biomedical engineering, following the ISO 10993–5 standard.

MCSs were seeded in a 96-well plate (2000 cells/well), and allowed to adhere for 24 hours. Then the cells were incubated with extracts of IVD tissue from 5 patients at three different concentrations: 10%, 50% and 100% for 48 hours. The assay was performed in 8 replicate measurements. The control group consisted of MSCs that were not incubated with extract. The incubation was stopped by removing the culture medium and adding culture medium supplemented with 0.5 mg/ml 3-(4,5-dimethylthiazol-2-yl)-2,5-diphenyltetrazolium bromide (MTT) (Sigma-Aldrich, Zwijndrecht, The Netherlands). After an additional incubation of 3 hours, the culture medium was carefully removed and 2-propanol (Merck, EMD Millipore, Darmstadt, Germany) was added. The 96-well plate was shaken for 15 minutes andabsorbance was read at 570 nm using the fluorostar Optima plate reader (BMG Labtech, De Meern, The Netherlands).

### Influence of IVD tissue extracts on osteoblast activity

In order to establish whether the extracts of IVD tissue would affect the ability of osteoblasts to mineralize their environment, the deposition of calcium by these cells in the extracellular matrix (ECM) was measured in samples with and without disc tissue extracts using the Alizarin Red S staining quantification assay (Sciencell Inc, Carlsbad, CA, USA; ARed-Q, Catalog #8678, 100 Tests) as described by the manufacturer. Disc tissue extracts (3% and 33%) from two patients were added to cultures of primary osteoblasts at 60% confluence for 48 hours, after which the Alizarin red S assay was performed. Controls were osteoblast cultures to which no extract was added. Next to measuring the amount of calcium deposits in the ECM, we also checked the confluence of the osteoblast cultures to ensure that the density of the osteoblast cultures was comparable with the control group.

### Influence of IVD extracts on osteogenic differentiation of MSCs

In order to evaluate the influence of disc tissue extracts on the osteogenic differentiation of MCSs, the deposition of calcium in the mineralized ECM by the differentiated MSCs was measured in samples with and without disc tissue extracts. After the MCSs reached 50–60% confluence and were incubated for 24 hours in an osteogenic differentiation medium, disc tissue extracts from two patients were added at concentrations of 12% and 1.2% in a 6-well plate. After 48 hours of incubation, the Alizarin Red S assay was performed. Two samples in osteogenic medium were used as control (osteogenic), as well as two samples in a proliferation medium (non osteogenic).

### Statistical analysis

Differences between samples in both MTT assay and Alizarin assay were tested for significance using Sigma plot 13 software. First, the data were tested for normal distribution with a Shapiro-Wilk test, followed by a equal variance test (Brown-Forsythe).

For all our data one of these tests failed, so the data were analyzed with a Kruskall Wallis ANOVA on ranks; followed by a Dunn’s (not normally distributed) or Student-Newman-Keuls (unequal variance) post hoc test.

## Results

### Influence of IVD extracts on viability of MCSs

The MTT assay assesses the activity of the mitochondrial β-nicotinamide adenine dinucleotide phosphate (NADPH)-depentent cellular oxidoreductase enzymes in converting MTT into water-insoluble formazan. This assay is generally used as a measure for cellular activity, proliferation of cells, or more specific, metabolic activity. In this study we will further stick to cellular activity.

**Fig 1:**
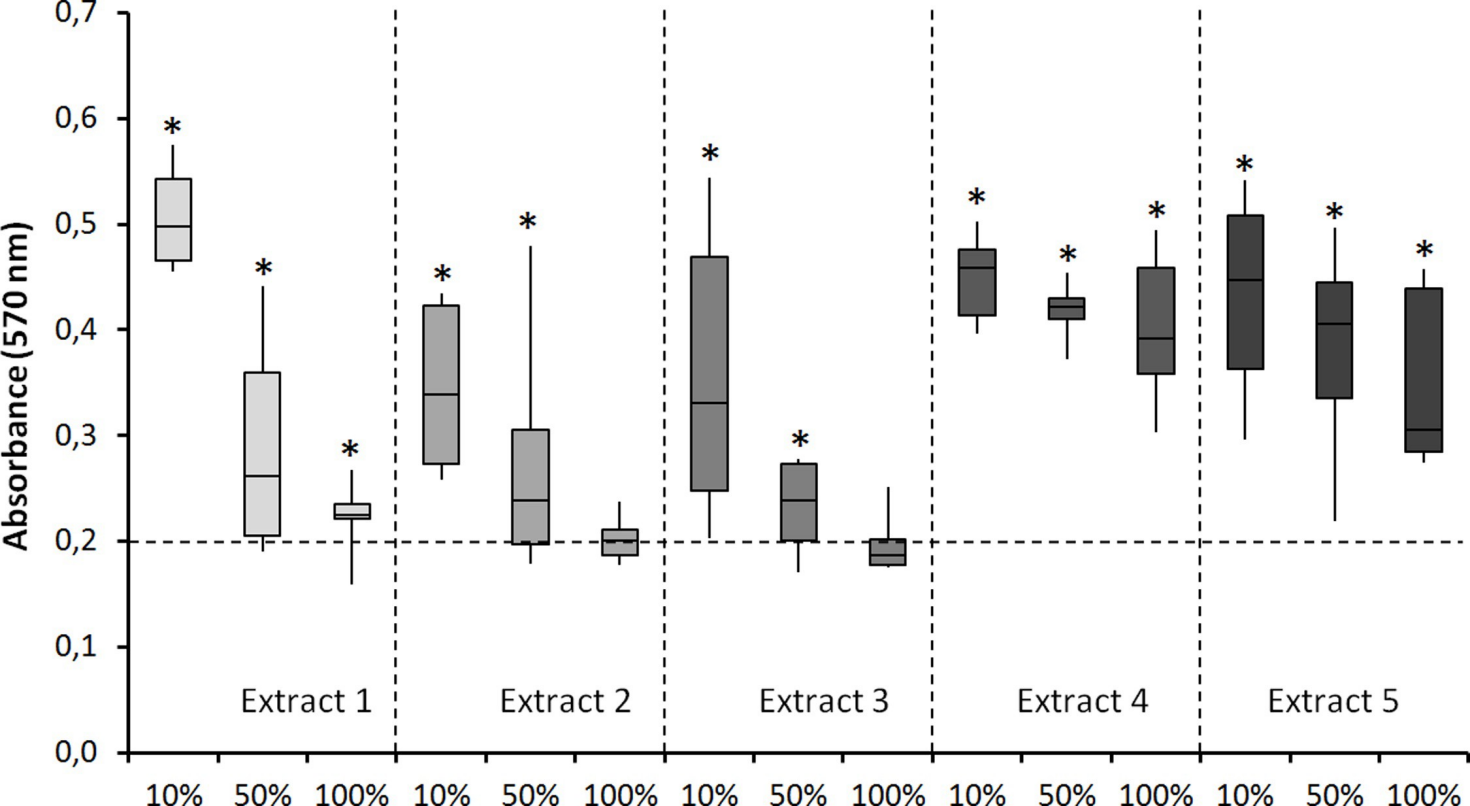
Effects of extracts of intervertebral disc tissue on the metabolic activity of human MSCs. Effects of extracts of IVD tissue from 5 different patients, applied at 3 concentrations on the metabolic activity of human MSCs, assessed using a MTT assay. The dashed horizontal line at 0.2 represents the mean of the control samples. All samples were tested in 8 fold. The * denotes a statistically significant difference (P<0.05) compared to the control samples.

Fig 1 shows the cellular activity of MSC which were exposed to extracts of IVD tissue at three different concentrations after 48 hours of incubation. Significantly higher levels of cellular activity were assessed in the group of MSCs co-cultured with extracts of IVD tissue compared to those of the control group (P = <0.001). This effect appeared to be inversely proportional to the concentration of IVD tissue extract. Significantly higher cellular activity was detected in MSCs incubated at 10% of IVD tissue extracts compared with the control group, those incubated at 50% of IVD tissue extracts in extracts 1, 2, and 3, and those incubated at 100% of extracts 1, 2, 3, and 5 (Table 2).

**Table 2:** Effects of extracts of intervertebral disc tissue on the metabolic activity of human MSCs. P-values after statistical analysis.

	Extract 1	Extract 2	Extract 3	Extract 4	Extract 5
Contr vs 10%	<0.001	<0.001	<0.001	<0.001	<0.001
Contr vs 50%	0.002	<0.001	0.004	<0.001	<0.001
Contr vs 100%	<0.001	ns	ns	<0.001	<0.001
10% vs 50%	<0.001	<0.001	<0.001	ns	ns
10% vs 100%	<0.001	<0.001	<0.001	ns	0.023
50% vs 100%	0.027	<0.001	0.003	ns	<0.001

Abbreviations: ns, non significant.

### Influence of IVD tissue extracts on osteoblast activity

**Fig 2:**
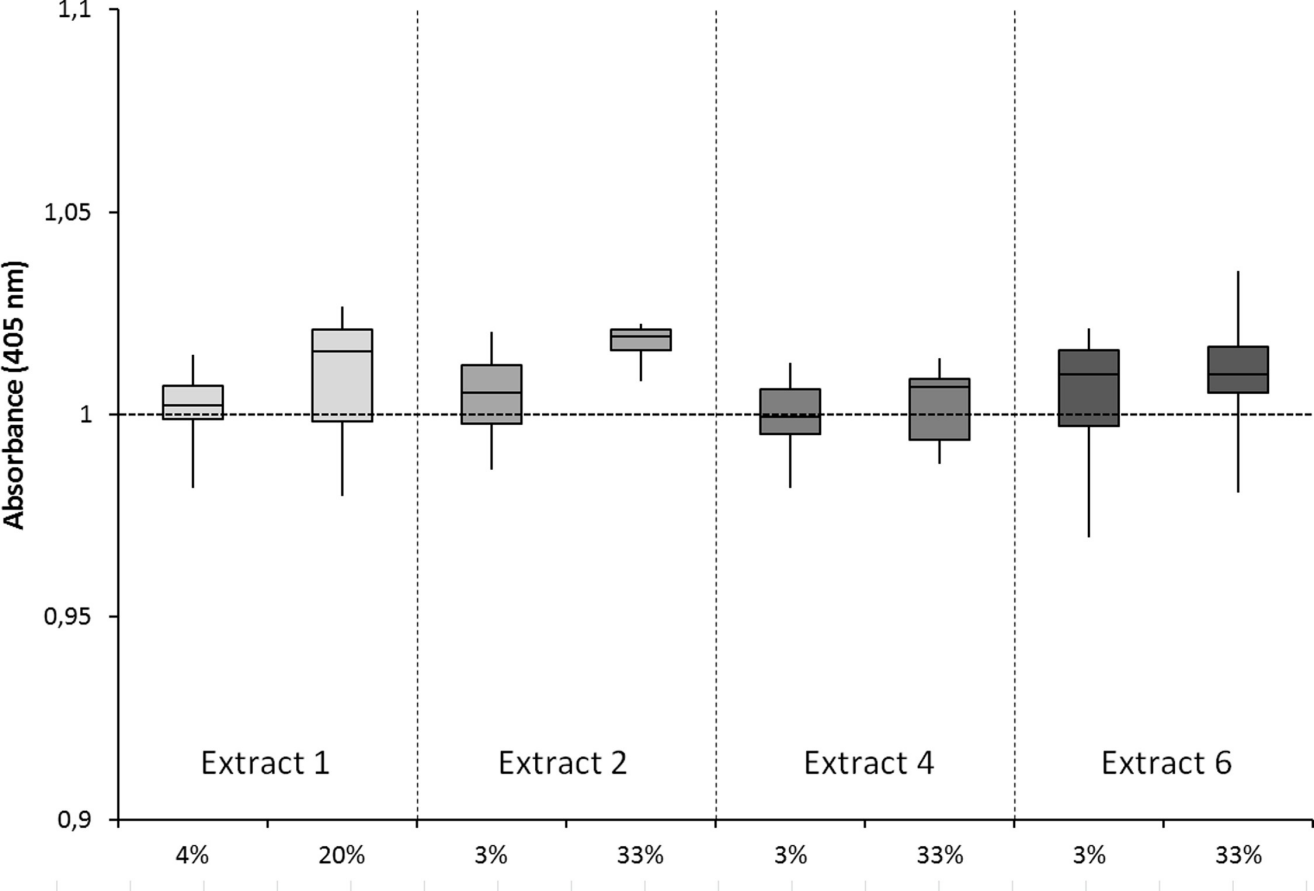
Alizarin Red assay quantification of obsteoblast activity. Effects of extracts of intervertebral disc tissue obtained from 4 different patients on mineralization by human osteoblasts using an Alizarin red S assay. No significant differences were obtained compared to the control (osteoblast without extract) set at 1.

Fig 2 shows the results of the Alizarin Red S assay of osteoblasts exposed to two different concentrations of extracts of IVD tissue after 48 hours of incubation. No significant differences in the amount of deposited calcium by human osteoblasts were established between the samples incubated with IVD tissue extracts at 3%, at 33% and the control sample. All osteoblast cultures had a similar confluency.

### Influence of IVD tissue extracts on osteogenic differentiation of MSCs

**Fig 3:**
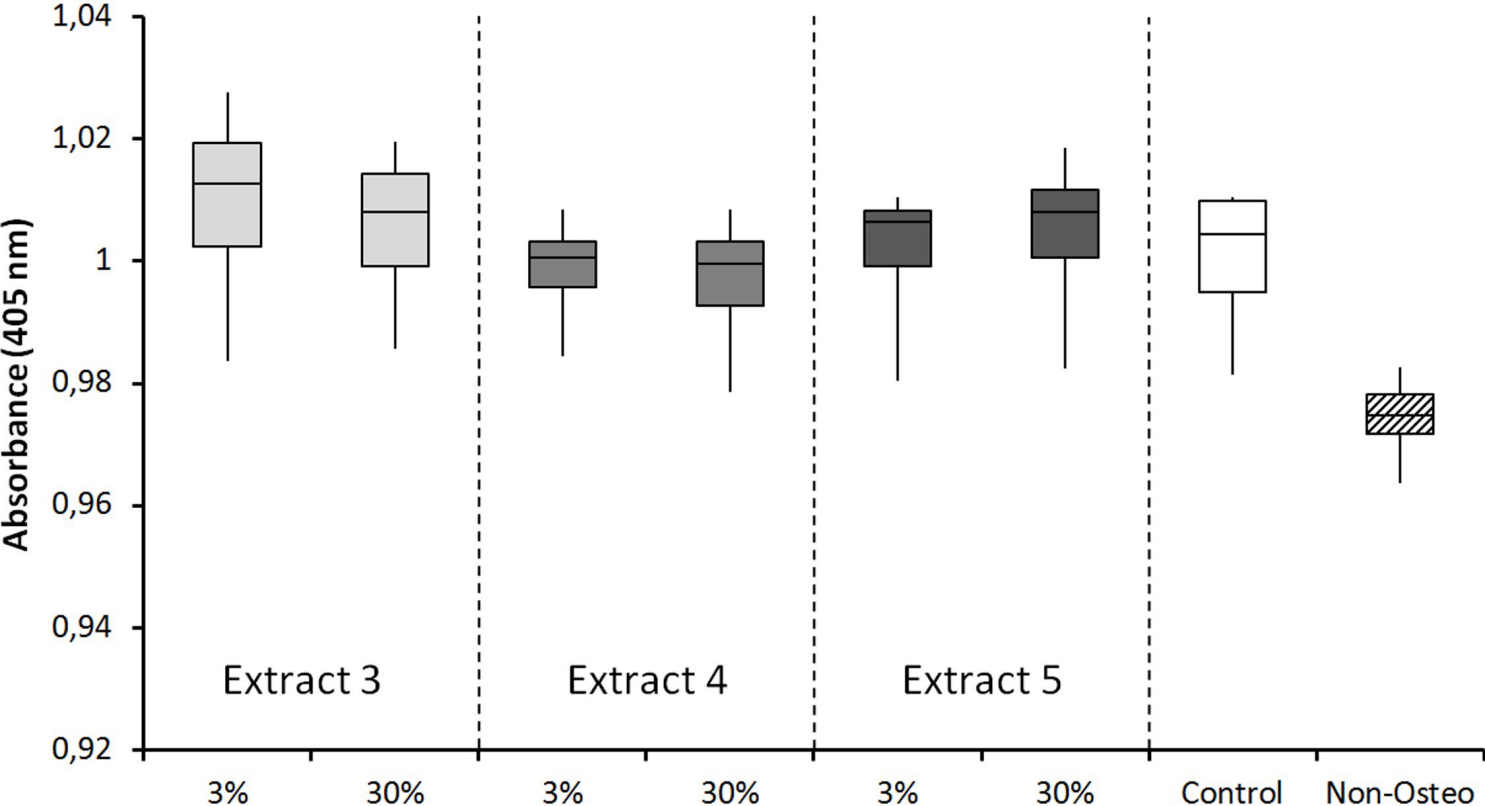
Alizarin red assay quantification of human MCSs osteogenesis. Effects of extracts of IVD tissues from three different patients, applied at two, indicated concentration, on human MSCs in osteogenic medium, using an Alizarin red S assay. No significant differences were obtained compared to the control MSCs in osteogenic medium without extract. MSCs in non-osteogenic medium (non-osteo) produced less mineral.

Fig 3 shows the results of the Alizarin Red S assay of MSCs. No significant differences were found in the amount of deposited calcium by the differentiated MSCs, the MSCs incubated with IVD tissue extracts (3 and 33%) and the control samples. The confluency of cells cultured in osteogenic medium of the three patients and the control group were comparable with each other. As expected, MSCs in non-osteogenic medium produced less (no) mineral.

## Discussion

The aims of this *in vitro* study were to evaluate the influence of IVD tissue extracts on (i) the cellular activity of human MCSs, (ii) their osteogenic differentiation, and (iii) the mineral production of osteblasts. The results of this study indicate that IVD tissue extracts might stimulate the cellular activity of MSCs, when present in low concentrations. The results also indicate that IVD tissue extracts have no influence on the osteogenic differentiation of MSCs, and on the mineralizing capacity of mature primary osteoblasts.

The increase in cellular activity of MSCs in the presence of low concentrations of IVD tissue extract is most probably cytokine mediated[9,10]. The positive influence of cytokines TNF-α,interleukins, IFN-γ onmitochondrial- and NADPH oxidase-generated reactive oxygen species production has been decribed before[11]. We did not observe inhibition of cellular activity of MSCs (compared to control condition) in the presence of any concentration of IVD tissue extract. From these observations, we conclude that IVD tissue extracts do not impede with MSC activity.

The *in vitro* study of Li et al. (2000) also evaluated the influence of IVD tissue extracts on the metabolism of osteoblast-like cells, and found that osteoblast proliferation as well as maturation was stimulated when IVD tissue was applied to osteoblasts in culture. Their results showed a stimulation of alkaline phosphatase production (maturation), cell proliferation measured by [3H]thymidine incorporation, and collagen type I production[12]. Chan et al. (2015) described, on the contrary, that primary IVD tissue cells inhibit osteogenesis of MSCs. In their study the incubation of MSCs with IVD tissue cells was maintained for 21 days, and resulted in a reduction of calcium deposition as observed by reduced alizarin red staining. A reduction in alkaline phosphatase activity in cocultures of MSCs with NP cells and RT-PCR analyses confirmed these results[13]. A possible explanation of the conflicting results with current study could be the differences in length of incubation time and concentration of IVD tissue material.

Li et al. (2002) conducted an *in vivo* study on pigs analysing the influence of IVD tissue on anterior spinal interbody fusion. They compared the bone fusion rate between the lumbar spine level with an implantation of Brantigan cage filled with a mixture of autograft and the NP tissue harvested from the removed disc level, and the spine level with a cage filled with autologous iliac crest bone graft in equal amounts. After 12 weeks CT evaluation showed that the level with NP tissue had a 20% fusion rate, while the level with pure autograft had a 70% fusion rate (P = 0.07). In their conclusion they stated that NP tissue mixed with autologous bone graft can cause a delay or decrease in bone formtion inside the cage[4]. A possible explanation of the conflicting results with current study could be again the amount of IVD tissue material, but also the use of healthy IVD tissue. In our study the extracts of diseased IVD tissue were used, which might cause differences in inflammatory cytokines releases.

The results of our study indicated that IVD tissue extracts might stimulate the viability of MSCs when they are present in low concentrations, but higher concentrations of IVD tissue extracts seemed to result in lower metabolic activity of MSCs. We consider it likely that this effect is caused by the more optimal concentrations of the effect-producing cytokines in the solution with the lower concentration of the extracts. Gabeen et al. (2014) show the stimulating effect of IL-4 at low concentration and a strong inhibiting effect at high concentrations[14].

Inflammation is the process by which the body tries to heal damaged tissue. Damaged IVD tissue will thus contain inflammatory cells and cytokines. In our study we used IVD tissue from patients who underwent PLIF surgery, which we considered to be inflamed tissue, containing inflammatory cells and cytokines, such as TNF-α, IL-4, -6,-12 and interferon-γ[15]. In spinal fusion surgery, remnants of inflamed NP en AF tissue will be a source of cytokines and chemokines which could interfere with the bone forming process[16]. Considering that the degree of inflammation varied in the samples of herniated disc tissue that were collected in the operating room, and that during the extraction period many cells underwent necrosis also releasing cytokines and chemokines, an excessive amount of cytokine and chemokine release could lead to unwanted effects *in vivo* and make these *in vitro* tests less representative. The number of cytokines which affected the results of this study is namely considered to be small compared to the number of cytokines in the human body. Therefore, it remains hard to predict what the effect of remaining IVD material will be on bone fusion *in vivo*. Future studies should include in vivo studies, investigating the effect of different amounts of remaining IVD tissue on spinal fusion, using an animal model with an already inflamed IVD. The involving cytokines could then be observed by intensive histological analyses and ELISA measurement.

Limitations of our study were the fact that a complete fusion environment could not be reproduced *in vitro*, as decribed above, and that we were also not able to use the same dilutions of tissue extracts for our experiments.

In conclusion, remaining disc material is not specific inhibiting the viability of MSCs when they are present in low concentrations. Even more, it might have more of a stimulating effect. Even if inflammatory cytokines are produced, these do not result in a net inhibition of cellular activity and osteogenic differentiation of MSCs and in the osteoblast metabolism.

## Supporting information

S1 File Description of isolation, culture and characterization of MSCs. (DOCX)

S2 File Supplementary information Fig 1, hMSC MTT. (XLSX)

S3 File Supplementary information Fig 2, hOB alizarin. (XLSX)

S4 File Supplementary information Fig 3, hMSC alizarin. (XLSX)

S5 File MTT-MSC individual patient extracts. (PDF)

S6 File MTT-MSC mean of all patients. (PDF)

S7 File Alizarin red S_MSC-3 patient extracts. (PDF)

S8 File Alizarin red S_hOB all patients. (PDF)

S9 File Alizarin red S_MSCs_all patients extracts. (PDF)
