# Supplementary material for: Is remaining intervertebral disc tissue interfering with bone generation during fusion of two vertebrae?
Source: PLoS One. 2019 Apr 25;14(4):e0215536. doi: 10.1371/journal.pone.0215536 (PMC6483188; doi:10.1371/journal.pone.0215536)
Supplement: S1 File — (DOCX) [file pone.0215536.s001.docx]

Supplementary data

From Buizer AT, Veldhuizen AG, Bulstra SK, Kuijer R. Static versus vacuum cell seeding on high and low porosity ceramic scaffolds. Journal of biomaterials applications. 2014 Jul;29(1):3-13 :

‘Isolation and culture of MSCs:

Bone marrow was acquired from male and female donors (88 total), undergoing total hip or knee surgery. The reaming debris from the femur was collected using a Jamshidi bone marrow aspiration needle (Care Fusion, McGaw Park, IL, USA). The study protocol was approved by the ethics committee of the University Medical Center Groningen. For each bone marrow sample four portions of 3ml were taken. Each portion was diluted with 5ml of phosphate-buffered saline (PBS) and subsequently processed by centrifugation over a density gradient (Histopaque 1077). Further processing took place according to the manufacturer’s instructions. After isolation, mononuclear cells were seeded in T75 tissue culture flasks (Greiner Bio-one, Alphen aan den Rijn, the

Netherlands) and cultured in minimal essential medium-alpha (a-MEM) supplemented with 10% heat-inactivated FBS, 0.1mM ascorbic acid-2-phosphate (AA2P, Fluka, Steinheim, Germany) and 2% Antibiotic-Antimycotic. The cells were grown in a humidified atmosphere with 5% CO_2_ at 37°C. Medium was changed twice a week. At 70% confluence, the cells were harvested and frozen in liquid nitrogen in full culture medium containing 7.5% DMSO (passage 1). Cells were thawed and cultured under 2D conditions until passage two before they were used in experiments.

Characterization of MSCs

hMSC antigen expression profile was assessed using a BD Stemflow Human MSC Analysis Kit (BD Biosciences, San Diego, CA, USA) according to the manufacturer’s instructions on a FACS machine (FACSCalibur, Beckton Dickinson, Franklin Lakes, NJ, USA). FACS data were analysed using the Kaluza software package (Beckman Coulter, Brea, CA, USA). Differentiation potential of the isolated cells was assessed by differentiation into osteogenic, adipogenic and chondrogenic cells. For osteogenic differentiation, 7500 cells per well were plated in a 24-well tissue culture plate (Greiner Bio-one, Alphen aan den Rijn, the Netherlands). The cells were incubated with osteogenic medium (a-MEM, 2% Antibiotic-Antimycotic, 10% heat inactivated FBS, 100nM dexamethasone, 0.05mM β-glycerophosphate and 0.05mM AA2P). After two weeks of culture, an alkaline phosphatase assay (Leukocyte alkaline phosphatase kit, Sigma, Steinheim, Germany) was performed according to the manufacturer’s instructions. For adipogenic differentiation, 10,000 cells per well were plated in a 24-well tissue culture plate. The cells were incubated with adipogenic medium (a-MEM, 2% Antibiotic-Antimycotic, 10% heat inactivated FBS, 1 mM dexamethasone, 0.5mM IBMX, 60 mM indomethacin and 10 mM human insulin) for three weeks. After this culture period, the cells were fixed in 3.7% paraformaldehyde (Boom, Meppel, the Netherlands). A 0.3 weight percentage Oil Red O stock solution in 99% 2-propanol was diluted 1.67 times in water and used for staining the cells. For chondrogenic differentiation, cell pellets of 250,000 cells were incubated with chondrogenic medium (a-MEM, 2% Antibiotic-Antimycotic, 0.1mM AA2P, 0.1 mM dexamethasone, 1% ITS+, 40 mg/ml L-proline and 10 ng/ml TGF-b1 (R&D Systems, Minneapolis, MN, USA)) for two weeks. After two weeks, the cell pellets were fixated in 3.7% paraformaldehyde and embedded in paraffin. Sections of 5 μm were deparaffinised, stained with a 1% Alcian blue (Boom, Meppel, the Netherlands) in 0.1M HCl solution and counterstained with a 0.2 weight percentage Nuclear Fast Red (Merck, Darmstadt, Germany) staining solution.’
