# Supplementary material for: Is remaining intervertebral disc tissue interfering with bone generation during fusion of two vertebrae?
Source: PLoS One. 2019 Apr 25;14(4):e0215536. doi: 10.1371/journal.pone.0215536 (PMC6483188; doi:10.1371/journal.pone.0215536)
Supplement: S5 File — (PDF) [file pone.0215536.s005.pdf]

One Way Analysis of Variance woensdag, mei 31, 2017, 10:02:51

Data source: Data 1 in Notebook1

Normality Test (Shapiro-Wilk): Passed (P = 0,941)

Equal Variance Test (Brown-Forsythe): Failed (P < 0,050)

Test execution ended by user request, ANOVA on Ranks begun

Kruskal-Wallis One Way Analysis of Variance on Ranks woensdag, mei 31, 2017, 10:02:51

Data source: Data 1 in Notebook1

Group	N	Missing	Median	25%	75%
P1 (10%)	8	0	0,298	0,259	0,352
P2 (10%)	8	0	0,139	0,0670	0,230
P3 (10%)	8	0	0,131	0,0412	0,298
P4 (10%)	8	0	0,144	0,0867	0,172
P5 (10%)	8	0	0,133	0,0115	0,206
P1 (50%)	8	0	0,0615	-0,00500	0,179
P2 (50%)	8	0	0,139	0,0670	0,230
P3 (50%)	8	0	0,0390	-0,00325	0,0757
P4 (50%)	8	0	0,107	0,0838	0,122
P5 (50%)	8	0	0,0905	-0,0420	0,138
P1 (100%)	8	0	0,0245	0,0178	0,0522
P2 (100%)	8	0	0,001000	-0,0170	0,0245
P3 (100%)	8	0	-0,0135	-0,0243	0,00925
P4 (100%)	8	0	0,0765	0,0153	0,166
P5 (100%)	8	0	-0,00950	-0,0335	0,125

H = 60,110 with 14 degrees of freedom. (P = <0,001)

The differences in the median values among the treatment groups are greater than would be expected by chance; there is a statistically significant difference (P = <0,001)

To isolate the group or groups that differ from the others use a multiple comparison procedure.

All Pairwise Multiple Comparison Procedures (Student-Newman-Keuls Method) :

Comparison	Diff of Ranks	q	P	P<0,050
P1 (10%) vs P3 (100%)	764,000	7,765	<0,001	Yes
P1 (10%) vs P2 (100%)	718,500	7,822	<0,001	Yes
P1 (10%) vs P1 (100%)	632,000	7,407	<0,001	Yes
P1 (10%) vs P5 (100%)	625,500	7,939	<0,001	Yes
P1 (10%) vs P3 (50%)	594,000	8,220	<0,001	Yes
P1 (10%) vs P5 (50%)	479,500	7,295	<0,001	Yes
P1 (10%) vs P1 (50%)	454,500	7,678	<0,001	Yes
P1 (10%) vs P4 (100%)	434,000	8,241	<0,001	Yes
P1 (10%) vs P5 (10%)	366,000	7,934	<0,001	Yes
P1 (10%) vs P4 (50%)	358,000	9,041	<0,001	Yes
P1 (10%) vs P3 (10%)	322,000	9,738	<0,001	Yes
P1 (10%) vs P2 (50%)	278,500	10,496	<0,001	Yes

P1 (10%) vs P2 (10%)	278,500	13,925	<0,001	Yes
P1 (10%) vs P4 (10%)	265,000	19,679	<0,001	Yes
P4 (10%) vs P3 (100%)	499,000	5,432	0,008	Yes
P4 (10%) vs P2 (100%)	453,500	5,315	0,010	Yes
P4 (10%) vs P1 (100%)	367,000	4,658	0,045	Yes
P4 (10%) vs P5 (100%)	360,500	4,989	0,017	Yes
P4 (10%) vs P3 (50%)	329,000	5,006	0,014	Yes
P4 (10%) vs P5 (50%)	214,500	3,624	0,202	No
P4 (10%) vs P1 (50%)	189,500	3,598	0,177	Do Not Test
P4 (10%) vs P4 (100%)	169,000	3,664	0,129	Do Not Test
P4 (10%) vs P5 (10%)	101,000	2,551	0,463	Do Not Test
P4 (10%) vs P4 (50%)	93,000	2,813	0,271	Do Not Test
P4 (10%) vs P3 (10%)	57,000	2,148	0,426	Do Not Test
P4 (10%) vs P2 (50%)	13,500	0,675	0,882	Do Not Test
P4 (10%) vs P2 (10%)	13,500	1,003	0,478	Do Not Test
P2 (10%) vs P3 (100%)	485,500	5,690	0,003	Yes
P2 (10%) vs P2 (100%)	440,000	5,584	0,003	Yes
P2 (10%) vs P1 (100%)	353,500	4,892	0,022	Yes
P2 (10%) vs P5 (100%)	347,000	5,279	0,006	Yes
P2 (10%) vs P3 (50%)	315,500	5,330	0,005	Yes
P2 (10%) vs P5 (50%)	201,000	3,817	0,123	Do Not Test
P2 (10%) vs P1 (50%)	176,000	3,815	0,099	Do Not Test
P2 (10%) vs P4 (100%)	155,500	3,927	0,061	Do Not Test
P2 (10%) vs P5 (10%)	87,500	2,646	0,333	Do Not Test
P2 (10%) vs P4 (50%)	79,500	2,996	0,147	Do Not Test
P2 (10%) vs P3 (10%)	43,500	2,175	0,273	Do Not Test
P2 (10%) vs P2 (50%)	0,000	0,000	1,000	Do Not Test
P2 (50%) vs P3 (100%)	485,500	6,162	<0,001	Yes
P2 (50%) vs P2 (100%)	440,000	6,089	<0,001	Yes
P2 (50%) vs P1 (100%)	353,500	5,378	0,005	Yes
P2 (50%) vs P5 (100%)	347,000	5,862	<0,001	Yes
P2 (50%) vs P3 (50%)	315,500	5,991	<0,001	Yes
P2 (50%) vs P5 (50%)	201,000	4,357	0,034	Do Not Test
P2 (50%) vs P1 (50%)	176,000	4,445	0,021	Do Not Test
P2 (50%) vs P4 (100%)	155,500	4,703	0,008	Do Not Test
P2 (50%) vs P5 (10%)	87,500	3,298	0,091	Do Not Test
P2 (50%) vs P4 (50%)	79,500	3,975	0,014	Do Not Test
P2 (50%) vs P3 (10%)	43,500	3,230	0,022	Do Not Test
P3 (10%) vs P3 (100%)	442,000	6,117	<0,001	Yes
P3 (10%) vs P2 (100%)	396,500	6,033	<0,001	Yes
P3 (10%) vs P1 (100%)	310,000	5,237	0,006	Yes
P3 (10%) vs P5 (100%)	303,500	5,763	0,001	Yes
P3 (10%) vs P3 (50%)	272,000	5,896	<0,001	Yes
P3 (10%) vs P5 (50%)	157,500	3,977	0,056	Do Not Test
P3 (10%) vs P1 (50%)	132,500	4,007	0,037	Do Not Test
P3 (10%) vs P4 (100%)	112,000	4,221	0,015	Do Not Test
P3 (10%) vs P5 (10%)	44,000	2,200	0,265	Do Not Test
P3 (10%) vs P4 (50%)	36,000	2,673	0,059	Do Not Test
P4 (50%) vs P3 (100%)	406,000	6,177	<0,001	Yes
P4 (50%) vs P2 (100%)	360,500	6,090	<0,001	Yes
P4 (50%) vs P1 (100%)	274,000	5,203	0,006	Yes
P4 (50%) vs P5 (100%)	267,500	5,799	<0,001	Yes
P4 (50%) vs P3 (50%)	236,000	5,960	<0,001	Yes
P4 (50%) vs P5 (50%)	121,500	3,675	0,071	Do Not Test
P4 (50%) vs P1 (50%)	96,500	3,637	0,050	Do Not Test
P4 (50%) vs P4 (100%)	76,000	3,800	0,020	Do Not Test

P4 (50%) vs P5 (10%)	8,000	0,594	0,674	Do Not Test
P5 (10%) vs P3 (100%)	398,000	6,724	<0,001	Yes
P5 (10%) vs P2 (100%)	352,500	6,694	<0,001	Yes
P5 (10%) vs P1 (100%)	266,000	5,766	0,001	Yes
P5 (10%) vs P5 (100%)	259,500	6,553	<0,001	Yes
P5 (10%) vs P3 (50%)	228,000	6,895	<0,001	Yes
P5 (10%) vs P5 (50%)	113,500	4,278	0,013	Do Not Test
P5 (10%) vs P1 (50%)	88,500	4,425	0,005	Do Not Test
P5 (10%) vs P4 (100%)	68,000	5,050	<0,001	Do Not Test
P4 (100%) vs P3 (100%)	330,000	6,266	<0,001	Yes
P4 (100%) vs P2 (100%)	284,500	6,167	<0,001	Yes
P4 (100%) vs P1 (100%)	198,000	5,000	0,006	Yes
P4 (100%) vs P5 (100%)	191,500	5,792	<0,001	Yes
P4 (100%) vs P3 (50%)	160,000	6,030	<0,001	Yes
P4 (100%) vs P5 (50%)	45,500	2,275	0,242	Do Not Test
P4 (100%) vs P1 (50%)	20,500	1,522	0,282	Do Not Test
P1 (50%) vs P3 (100%)	309,500	6,709	<0,001	Yes
P1 (50%) vs P2 (100%)	264,000	6,667	<0,001	Yes
P1 (50%) vs P1 (100%)	177,500	5,368	0,001	Yes
P1 (50%) vs P5 (100%)	171,000	6,445	<0,001	Yes
P1 (50%) vs P3 (50%)	139,500	6,975	<0,001	Yes
P1 (50%) vs P5 (50%)	25,000	1,857	0,189	Do Not Test
P5 (50%) vs P3 (100%)	284,500	7,185	<0,001	Yes
P5 (50%) vs P2 (100%)	239,000	7,228	<0,001	Yes
P5 (50%) vs P1 (100%)	152,500	5,748	<0,001	Yes
P5 (50%) vs P5 (100%)	146,000	7,300	<0,001	Yes
P5 (50%) vs P3 (50%)	114,500	8,503	<0,001	Yes
P3 (50%) vs P3 (100%)	170,000	5,141	0,003	Yes
P3 (50%) vs P2 (100%)	124,500	4,692	0,005	Yes
P3 (50%) vs P1 (100%)	38,000	1,900	0,371	No
P3 (50%) vs P5 (100%)	31,500	2,339	0,098	Do Not Test
P5 (100%) vs P3 (100%)	138,500	5,220	0,001	Yes
P5 (100%) vs P2 (100%)	93,000	4,650	0,003	Yes
P5 (100%) vs P1 (100%)	6,500	0,483	0,733	Do Not Test
P1 (100%) vs P3 (100%)	132,000	6,600	<0,001	Yes
P1 (100%) vs P2 (100%)	86,500	6,424	<0,001	Yes
P2 (100%) vs P3 (100%)	45,500	3,379	0,017	Yes

Note: The multiple comparisons on ranks do not include an adjustment for ties.

A result of "Do Not Test" occurs for a comparison when no significant difference is found between the two rank sums that enclose that comparison. For example, if you had four rank sums sorted in order, and found no significant difference between rank sums 4 vs. 2, then you would not test 4 vs. 3 and 3 vs. 2, but still test 4 vs. 1 and 3 vs. 1 (4 vs. 3 and 3 vs. 2 are enclosed by 4 vs. 2: 4 3 2 1). Note that not testing the enclosed rank sums is a procedural rule, and a result of Do Not Test should be treated as if there is no significant difference between the rank sums, even though one may appear to exist.
