# Supplementary material for: Is remaining intervertebral disc tissue interfering with bone generation during fusion of two vertebrae?
Source: PLoS One. 2019 Apr 25;14(4):e0215536. doi: 10.1371/journal.pone.0215536 (PMC6483188; doi:10.1371/journal.pone.0215536)
Supplement: S6 File — (PDF) [file pone.0215536.s006.pdf]

Mean of all patient extracts at three different concentrations

One Way Analysis of Variance woensdag, mei 31, 2017, 10:13:50

Data source: Data 1 in Stat-analysis_Zahrina

Normality Test (Shapiro-Wilk): Passed (P = 0,173)

Equal Variance Test (Brown-Forsythe): Failed (P < 0,050)

Test execution ended by user request, ANOVA on Ranks begun

Kruskal-Wallis One Way Analysis of Variance on Ranks woensdag, mei 31, 2017, 10:13:50

Data source: Data 1 in Stat-analysis_Zahrina

Group	N	Missing	Median	25%	75%
10%	40	0	0,163	0,0653	0,255
50%	40	0	0,0775	0,0517	0,136
100%	40	0	0,00900	-0,0190	0,0603

H = 35,714 with 2 degrees of freedom. (P = <0,001)

The differences in the median values among the treatment groups are greater than would be expected by chance; there is a statistically significant difference (P = <0,001)

To isolate the group or groups that differ from the others use a multiple comparison procedure.

All Pairwise Multiple Comparison Procedures (Student-Newman-Keuls Method) :

Comparison	Diff of Ranks	q	P	P<0,050
10% vs 100%	1855,500	8,434	<0,001	Yes
10% vs 50%	826,500	5,624	<0,001	Yes
50% vs 100%	1029,000	7,001	<0,001	Yes

Note: The multiple comparisons on ranks do not include an adjustment for ties.
