# Supplementary material for: Is remaining intervertebral disc tissue interfering with bone generation during fusion of two vertebrae?
Source: PLoS One. 2019 Apr 25;14(4):e0215536. doi: 10.1371/journal.pone.0215536 (PMC6483188; doi:10.1371/journal.pone.0215536)
Supplement: S7 File — (PDF) [file pone.0215536.s007.pdf]

**One Way Analysis of Variance** woensdag, mei 31, 2017, 10:17:18

**Data source:** Data 2 in Stat-analysis_Zahrina-2
Alizarin Red S - MSCs vs non-osteogenic.
3 patient extracts were tested in 2 different concentrations.

**Normality Test (Shapiro-Wilk):** Failed (P < 0,050)

Test execution ended by user request, ANOVA on Ranks begun

**Kruskal-Wallis One Way Analysis of Variance on Ranks** woensdag, mei 31, 2017, 10:17:18

**Data source:** Data 2 in Stat-analysis_Zahrina-2

| **Group** | **N** | **Missing** | **Median** | **25%** | **75%** |
|---|---|---|---|---|---|
| Control | 16 | 0 | 0,00406 | -0,00819 | 0,00806 |
| P4 30% | 8 | 0 | 0,00256 | -0,0177 | 0,00856 |
| P4 3% | 8 | 0 | 0,00106 | -0,0127 | 0,0136 |
| P3 30% | 4 | 0 | 0,0106 | -0,00744 | 0,0203 |
| P3 3% | 4 | 0 | 0,0151 | -0,00769 | 0,0273 |
| P7 30% | 4 | 0 | 0,00556 | -0,0139 | 0,0138 |
| P7 3% | 4 | 0 | 0,00406 | -0,0157 | 0,00731 |
| Non-osteo | 8 | 0 | -0,0229 | -0,0299 | -0,0184 |

H = 20,075 with 7 degrees of freedom. (P = 0,005)

The differences in the median values among the treatment groups are greater than would be expected by chance; there is a statistically significant difference (P = 0,005)

To isolate the group or groups that differ from the others use a multiple comparison procedure.

All Pairwise Multiple Comparison Procedures (Dunn's Method) :

| **Comparison** | **Diff of Ranks** | **Q** | **P** | **P<0,050** |
|---|---|---|---|---|
| P3 3% vs Non-osteo | 36,938 | 3,698 | 0,006 | Yes |
| P3 3% vs P7 3% | 16,250 | 1,409 | 1,000 | No |
| P3 3% vs P4 30% | 16,188 | 1,621 | 1,000 | Do Not Test |
| P3 3% vs P4 3% | 13,563 | 1,358 | 1,000 | Do Not Test |
| P3 3% vs Control | 13,469 | 1,477 | 1,000 | Do Not Test |
| P3 3% vs P7 30% | 11,125 | 0,965 | 1,000 | Do Not Test |
| P3 3% vs P3 30% | 4,125 | 0,358 | 1,000 | Do Not Test |
| P3 30% vs Non-osteo | 32,813 | 3,285 | 0,029 | Yes |
| P3 30% vs P7 3% | 12,125 | 1,051 | 1,000 | Do Not Test |
| P3 30% vs P4 30% | 12,063 | 1,208 | 1,000 | Do Not Test |
| P3 30% vs P4 3% | 9,438 | 0,945 | 1,000 | Do Not Test |
| P3 30% vs Control | 9,344 | 1,025 | 1,000 | Do Not Test |
| P3 30% vs P7 30% | 7,000 | 0,607 | 1,000 | Do Not Test |
| P7 30% vs Non-osteo | 25,813 | 2,584 | 0,273 | No |
| P7 30% vs P7 3% | 5,125 | 0,444 | 1,000 | Do Not Test |
| P7 30% vs P4 30% | 5,063 | 0,507 | 1,000 | Do Not Test |
| P7 30% vs P4 3% | 2,438 | 0,244 | 1,000 | Do Not Test |
| P7 30% vs Control | 2,344 | 0,257 | 1,000 | Do Not Test |
| Control vs Non-osteo | 23,469 | 3,323 | 0,025 | Do Not Test |

| | | | | |
|---|---|---|---|---|
| Control vs P7 3% | 2,781 | 0,305 | 1,000 | Do Not Test |
| Control vs P4 30% | 2,719 | 0,385 | 1,000 | Do Not Test |
| Control vs P4 3% | 0,0938 | 0,0133 | 1,000 | Do Not Test |
| P4 3% vs Non-osteo | 23,375 | 2,866 | 0,116 | Do Not Test |
| P4 3% vs P7 3% | 2,688 | 0,269 | 1,000 | Do Not Test |
| P4 3% vs P4 30% | 2,625 | 0,322 | 1,000 | Do Not Test |
| P4 30% vs Non-osteo | 20,750 | 2,545 | 0,306 | Do Not Test |
| P4 30% vs P7 3% | 0,0625 | 0,00626 | 1,000 | Do Not Test |
| P7 3% vs Non-osteo | 20,688 | 2,071 | 1,000 | Do Not Test |

Note: The multiple comparisons on ranks do not include an adjustment for ties.
