# Supplementary material for: Is remaining intervertebral disc tissue interfering with bone generation during fusion of two vertebrae?
Source: PLoS One. 2019 Apr 25;14(4):e0215536. doi: 10.1371/journal.pone.0215536 (PMC6483188; doi:10.1371/journal.pone.0215536)
Supplement: S8 File — (PDF) [file pone.0215536.s008.pdf]

One Way Analysis of Variance woensdag, mei 31, 2017, 10:56:12

Data source: Data 3 in Stat-analysis_Zahrina-2
HOB alizarin red S assay_all patients extracts

Normality Test (Shapiro-Wilk): Failed (P < 0,050)

Test execution ended by user request, ANOVA on Ranks begun

Kruskal-Wallis One Way Analysis of Variance on Ranks woensdag, mei 31, 2017, 10:56:12

Data source: Data 3 in Stat-analysis_Zahrina-2

Group	N	Missing	Median	25%	75%
p6 33%	12	0	0,00988	0,00663	0,0179
p6 3%	12	0	0,0159	0,00538	0,0201
p2 33%	4	0	0,0194	0,0109	0,0219
P2 3%	4	0	0,00538	-0,00987	0,0176
C1	8	0	-0,00775	-0,0222	-0,000250
P4 33%	5	0	0,00647	-0,00953	0,0110
P4 3%	8	0	-0,00103	-0,00803	0,00897
P6 33%	9	0	0,00547	-0,0135	0,0165
P6 3%	9	0	-0,00453	-0,0245	0,00547
C2	17	0	-0,0573	-0,0628	-0,0493
P1 20%	8	0	0,0158	-0,00675	0,0215
P1 4%	8	0	0,00225	-0,00325	0,00750
C3	8	0	-0,00225	-0,00550	0,00525

H = 64,478 with 12 degrees of freedom. (P = <0,001)

The differences in the median values among the treatment groups are greater than would be expected by chance; there is a statistically significant difference (P = <0,001)

To isolate the group or groups that differ from the others use a multiple comparison procedure.

All Pairwise Multiple Comparison Procedures (Dunn's Method) :

Comparison	Diff of Ranks	Q	P	P<0,050
p2 33% vs C2	85,243	4,723	<0,001	Yes
p2 33% vs C1	58,063	2,920	0,273	No
p2 33% vs P6 3%	53,569	2,745	0,472	Do Not Test
p2 33% vs P4 3%	41,875	2,106	1,000	Do Not Test
p2 33% vs C3	41,438	2,084	1,000	Do Not Test
p2 33% vs P1 4%	36,938	1,857	1,000	Do Not Test
p2 33% vs P6 33%	36,625	1,877	1,000	Do Not Test
p2 33% vs P4 33%	36,225	1,663	1,000	Do Not Test
p2 33% vs P2 3%	29,250	1,274	1,000	Do Not Test
p2 33% vs P1 20%	18,563	0,933	1,000	Do Not Test
p2 33% vs p6 33%	15,417	0,822	1,000	Do Not Test
p2 33% vs p6 3%	9,917	0,529	1,000	Do Not Test
p6 3% vs C2	75,326	6,152	<0,001	Yes
p6 3% vs C1	48,146	3,248	0,091	Do Not Test
p6 3% vs P6 3%	43,653	3,048	0,180	Do Not Test

p6 3% vs P4 3%	31,958	2,156	1,000	Do Not Test
p6 3% vs C3	31,521	2,126	1,000	Do Not Test
p6 3% vs P1 4%	27,021	1,823	1,000	Do Not Test
p6 3% vs P6 33%	26,708	1,865	1,000	Do Not Test
p6 3% vs P4 33%	26,308	1,522	1,000	Do Not Test
p6 3% vs P2 3%	19,333	1,031	1,000	Do Not Test
p6 3% vs P1 20%	8,646	0,583	1,000	Do Not Test
p6 3% vs p6 33%	5,500	0,415	1,000	Do Not Test
p6 33% vs C2	69,826	5,703	<0,001	Yes
p6 33% vs C1	42,646	2,877	0,313	Do Not Test
p6 33% vs P6 3%	38,153	2,664	0,602	Do Not Test
p6 33% vs P4 3%	26,458	1,785	1,000	Do Not Test
p6 33% vs C3	26,021	1,755	1,000	Do Not Test
p6 33% vs P1 4%	21,521	1,452	1,000	Do Not Test
p6 33% vs P6 33%	21,208	1,481	1,000	Do Not Test
p6 33% vs P4 33%	20,808	1,204	1,000	Do Not Test
p6 33% vs P2 3%	13,833	0,738	1,000	Do Not Test
p6 33% vs P1 20%	3,146	0,212	1,000	Do Not Test
P1 20% vs C2	66,680	4,789	<0,001	Yes
P1 20% vs C1	39,500	2,433	1,000	Do Not Test
P1 20% vs P6 3%	35,007	2,218	1,000	Do Not Test
P1 20% vs P4 3%	23,313	1,436	1,000	Do Not Test
P1 20% vs C3	22,875	1,409	1,000	Do Not Test
P1 20% vs P1 4%	18,375	1,132	1,000	Do Not Test
P1 20% vs P6 33%	18,063	1,145	1,000	Do Not Test
P1 20% vs P4 33%	17,663	0,954	1,000	Do Not Test
P1 20% vs P2 3%	10,688	0,537	1,000	Do Not Test
P2 3% vs C2	55,993	3,103	0,150	No
P2 3% vs C1	28,813	1,449	1,000	Do Not Test
P2 3% vs P6 3%	24,319	1,246	1,000	Do Not Test
P2 3% vs P4 3%	12,625	0,635	1,000	Do Not Test
P2 3% vs C3	12,188	0,613	1,000	Do Not Test
P2 3% vs P1 4%	7,688	0,387	1,000	Do Not Test
P2 3% vs P6 33%	7,375	0,378	1,000	Do Not Test
P2 3% vs P4 33%	6,975	0,320	1,000	Do Not Test
P4 33% vs C2	49,018	2,967	0,235	Do Not Test
P4 33% vs C1	21,837	1,180	1,000	Do Not Test
P4 33% vs P6 3%	17,344	0,958	1,000	Do Not Test
P4 33% vs P4 3%	5,650	0,305	1,000	Do Not Test
P4 33% vs C3	5,212	0,282	1,000	Do Not Test
P4 33% vs P1 4%	0,712	0,0385	1,000	Do Not Test
P4 33% vs P6 33%	0,400	0,0221	1,000	Do Not Test
P6 33% vs C2	48,618	3,632	0,022	Do Not Test
P6 33% vs C1	21,438	1,358	1,000	Do Not Test
P6 33% vs P6 3%	16,944	1,107	1,000	Do Not Test
P6 33% vs P4 3%	5,250	0,333	1,000	Do Not Test
P6 33% vs C3	4,813	0,305	1,000	Do Not Test
P6 33% vs P1 4%	0,313	0,0198	1,000	Do Not Test
P1 4% vs C2	48,305	3,469	0,041	Do Not Test
P1 4% vs C1	21,125	1,301	1,000	Do Not Test
P1 4% vs P6 3%	16,632	1,054	1,000	Do Not Test
P1 4% vs P4 3%	4,938	0,304	1,000	Do Not Test
P1 4% vs C3	4,500	0,277	1,000	Do Not Test
C3 vs C2	43,805	3,146	0,129	Do Not Test
C3 vs C1	16,625	1,024	1,000	Do Not Test
C3 vs P6 3%	12,132	0,769	1,000	Do Not Test

C3 vs P4 3%	0,438	0,0269	1,000	Do Not Test
P4 3% vs C2	43,368	3,115	0,144	Do Not Test
P4 3% vs C1	16,188	0,997	1,000	Do Not Test
P4 3% vs P6 3%	11,694	0,741	1,000	Do Not Test
P6 3% vs C2	31,673	2,366	1,000	Do Not Test
P6 3% vs C1	4,493	0,285	1,000	Do Not Test
C1 vs C2	27,180	1,952	1,000	Do Not Test

Note: The multiple comparisons on ranks do not include an adjustment for ties.
