# Supplementary material for: Is remaining intervertebral disc tissue interfering with bone generation during fusion of two vertebrae?
Source: PLoS One. 2019 Apr 25;14(4):e0215536. doi: 10.1371/journal.pone.0215536 (PMC6483188; doi:10.1371/journal.pone.0215536)
Supplement: S9 File — (PDF) [file pone.0215536.s009.pdf]

One Way Analysis of Variance woensdag, mei 31, 2017, 11:10:07

Data source: Data 4 in Stat-analysis_Zahrina-2
Alizarin red S, MSCs ostegenic medium vs non-osteogenic medium
extracts of alle patients tested, in two different concentrations

Normality Test (Shapiro-Wilk): Failed (P < 0,050)

Test execution ended by user request, ANOVA on Ranks begun

Kruskal-Wallis One Way Analysis of Variance on Ranks woensdag, mei 31, 2017, 11:10:07

Data source: Data 4 in Stat-analysis_Zahrina-2

**Group**	**N**	**Missing**	**Median**	**25%**	**75%**
P4 3%	4	0	0,000500	-0,0118	0,00675
P4 33%	4	0	-0,000500	-0,0168	0,00675
P5 3%	4	0	0,00650	-0,0133	0,00975
P5 33%	4	0	0,00800	-0,0115	0,0162
Control	24	0	0,00378	-0,00819	0,00806
P1 30%	8	0	0,00256	-0,0177	0,00856
P1 3%	8	0	0,00106	-0,0127	0,0136
P5 30%	4	0	0,0106	-0,00744	0,0203
P5 3%	4	0	0,0151	-0,00769	0,0273
P3 30%	4	0	0,00556	-0,0139	0,0138
P3 3%	4	0	0,00406	-0,0157	0,00731
Non-osteo	8	0	-0,0229	-0,0299	-0,0184

H = 23,358 with 11 degrees of freedom. (P = 0,016)

The differences in the median values among the treatment groups are greater than would be expected by chance; there is a statistically significant difference (P = 0,016)

To isolate the group or groups that differ from the others use a multiple comparison procedure.

All Pairwise Multiple Comparison Procedures (Dunn's Method) :

**Comparison**	**Diff of Ranks**	**Q**	**P**	**P<0,050**
P5 3% vs Non-osteo	54,813	3,852	0,008	Yes
P5 3% vs P4 33%	28,500	1,734	1,000	No
P5 3% vs P1 30%	24,938	1,752	1,000	Do Not Test
P5 3% vs P3 3%	24,750	1,506	1,000	Do Not Test
P5 3% vs P4 3%	24,375	1,483	1,000	Do Not Test
P5 3% vs P1 3%	20,938	1,471	1,000	Do Not Test
P5 3% vs Control	20,563	1,638	1,000	Do Not Test
P5 3% vs P3 30%	17,125	1,042	1,000	Do Not Test
P5 3% vs P5 3%	16,125	0,981	1,000	Do Not Test
P5 3% vs P5 33%	10,500	0,639	1,000	Do Not Test
P5 3% vs P5 30%	6,375	0,388	1,000	Do Not Test
P5 30% vs Non-osteo	48,438	3,404	0,044	Yes
P5 30% vs P4 33%	22,125	1,346	1,000	Do Not Test
P5 30% vs P1 30%	18,563	1,304	1,000	Do Not Test
P5 30% vs P3 3%	18,375	1,118	1,000	Do Not Test
P5 30% vs P4 3%	18,000	1,095	1,000	Do Not Test

P5 30% vs P1 3%	14,563	1,023	1,000	Do Not Test
P5 30% vs Control	14,188	1,130	1,000	Do Not Test
P5 30% vs P3 30%	10,750	0,654	1,000	Do Not Test
P5 30% vs P5 3%	9,750	0,593	1,000	Do Not Test
P5 30% vs P5 33%	4,125	0,251	1,000	Do Not Test
P5 33% vs Non-osteo	44,313	3,114	0,122	No
P5 33% vs P4 33%	18,000	1,095	1,000	Do Not Test
P5 33% vs P1 30%	14,438	1,015	1,000	Do Not Test
P5 33% vs P3 3%	14,250	0,867	1,000	Do Not Test
P5 33% vs P4 3%	13,875	0,844	1,000	Do Not Test
P5 33% vs P1 3%	10,438	0,733	1,000	Do Not Test
P5 33% vs Control	10,063	0,802	1,000	Do Not Test
P5 33% vs P3 30%	6,625	0,403	1,000	Do Not Test
P5 33% vs P5 3%	5,625	0,342	1,000	Do Not Test
P5 3% vs Non-osteo	38,688	2,719	0,433	Do Not Test
P5 3% vs P4 33%	12,375	0,753	1,000	Do Not Test
P5 3% vs P1 30%	8,813	0,619	1,000	Do Not Test
P5 3% vs P3 3%	8,625	0,525	1,000	Do Not Test
P5 3% vs P4 3%	8,250	0,502	1,000	Do Not Test
P5 3% vs P1 3%	4,813	0,338	1,000	Do Not Test
P5 3% vs Control	4,438	0,354	1,000	Do Not Test
P5 3% vs P3 30%	1,000	0,0609	1,000	Do Not Test
P3 30% vs Non-osteo	37,688	2,648	0,534	Do Not Test
P3 30% vs P4 33%	11,375	0,692	1,000	Do Not Test
P3 30% vs P1 30%	7,813	0,549	1,000	Do Not Test
P3 30% vs P3 3%	7,625	0,464	1,000	Do Not Test
P3 30% vs P4 3%	7,250	0,441	1,000	Do Not Test
P3 30% vs P1 3%	3,813	0,268	1,000	Do Not Test
P3 30% vs Control	3,438	0,274	1,000	Do Not Test
Control vs Non-osteo	34,250	3,610	0,020	Do Not Test
Control vs P4 33%	7,938	0,632	1,000	Do Not Test
Control vs P1 30%	4,375	0,461	1,000	Do Not Test
Control vs P3 3%	4,188	0,334	1,000	Do Not Test
Control vs P4 3%	3,813	0,304	1,000	Do Not Test
Control vs P1 3%	0,375	0,0395	1,000	Do Not Test
P1 3% vs Non-osteo	33,875	2,915	0,234	Do Not Test
P1 3% vs P4 33%	7,563	0,531	1,000	Do Not Test
P1 3% vs P1 30%	4,000	0,344	1,000	Do Not Test
P1 3% vs P3 3%	3,813	0,268	1,000	Do Not Test
P1 3% vs P4 3%	3,438	0,242	1,000	Do Not Test
P4 3% vs Non-osteo	30,438	2,139	1,000	Do Not Test
P4 3% vs P4 33%	4,125	0,251	1,000	Do Not Test
P4 3% vs P1 30%	0,563	0,0395	1,000	Do Not Test
P4 3% vs P3 3%	0,375	0,0228	1,000	Do Not Test
P3 3% vs Non-osteo	30,063	2,113	1,000	Do Not Test
P3 3% vs P4 33%	3,750	0,228	1,000	Do Not Test
P3 3% vs P1 30%	0,188	0,0132	1,000	Do Not Test
P1 30% vs Non-osteo	29,875	2,571	0,669	Do Not Test
P1 30% vs P4 33%	3,563	0,250	1,000	Do Not Test
P4 33% vs Non-osteo	26,313	1,849	1,000	Do Not Test

Note: The multiple comparisons on ranks do not include an adjustment for ties.
